# Degradation of T-Tubular Microdomains and Altered cAMP Compartmentation Lead to Emergence of Arrhythmogenic Triggers in Heart Failure Myocytes: An *in silico* Study

**DOI:** 10.3389/fphys.2018.01737

**Published:** 2018-12-04

**Authors:** Alexandra D. Loucks, Thomas O’Hara, Natalia A. Trayanova

**Affiliations:** Institute for Computational Medicine and Department of Biomedical Engineering at Johns Hopkins University, Baltimore, MD, United States

**Keywords:** heart failure, computational modeling, early afterdepolarizations, arrhythmia, microdomain degradation

## Abstract

Heart failure (HF) is one of the most common causes of morbidity and mortality worldwide. Although many patients suffering from HF die from sudden cardiac death caused by arrhythmias, the mechanism linking HF remodeling to an increased arrhythmogenic propensity remains incomplete. HF is typically characterized by a progressive loss of transverse tubule (T-tubule) domains, which leads to an altered distribution of L-type calcium channels (LTCCs). Microdomain degradation also causes the disruption of the β_2_ adrenergic receptor (β_2_AR) and phosphodiesterase (PDE) signaling localization, normally confined to the dyadic space. The goal of this study was to analyze how these subcellular changes affect the function of LTCCs and lead to the emergence of ventricular cell-level triggers of arrhythmias. To accomplish this, we developed a novel computational model of a human ventricular HF myocyte in which LTCCs were divided into six different populations, based on their location and signaling environment they experience. To do so, we included T-tubular microdomain remodeling which led to a subset of LTCCs to be redistributed from the T-tubular to the surface membrane and allowed for different levels of phosphorylation of LTCCs by PKA, based on the presence of β_2_ARs and PDEs. The model was used to study the behavior of the LTCC current (I_CaL_) under basal and sympathetic stimulation and its effect on cellular action potential. Our results showed that channels redistributed from the T-tubular membrane to the bulk of the sarcolemma displayed an altered function in their new, non-native signaling domain. Incomplete calcium dependent inactivation, which resulted in a longer-lasting and larger-in-magnitude LTCC current, was observed when we decoupled LTCCs from ryanodine receptors and removed them from the dyadic space. The magnitude of the LTCC current, especially in the surface sarcolemma, was also increased via phosphorylation by the redistributed β_2_ARs and PDEs. These changes in LTCC current led to the development of early afterdepolarizations. Thus, our study shows that altered LTCC function is a potential cause for the emergence of cell-level triggers of arrhythmia, and that β_2_ARs and PDEs present useful therapeutic targets for treatment of HF and prevention of sudden cardiac death.

## Introduction

The occurrence of heart failure (HF), a disease currently affecting about 5.7 million Americans, is expected to rise by 46% from 2012 to 2030 as the population ages (Mozaffarian et al., 2016). HF patients are 6 to 9 times more likely to die from sudden cardiac death due to lethal arrhythmias than healthy individuals (Tomaselli and Zipes, 2004). Despite these troubling statistics, the mechanism linking pathophysiological remodeling to arrhythmogenesis in HF patients remains poorly understood. This has resulted in ineffective pharmacological therapy for preventing sudden arrhythmic death and in unsuccessful approaches to arrhythmia risk stratification of HF patients (Stevenson, 2003; Fishman et al., 2010).

Heart failure causes the heart to undergo remodeling across multiple scales (Tomaselli and Marbán, 1999). However, studies have shown that nearly all arrhythmias in non-ischemic HF and approximately 50% of those in ischemic HF develop due to abnormal automaticity or triggered activity such as early afterdepolarizations (EADs) and delayed afterdepolarizations (Rubart and Zipes, 2005).

Previous research has also shown that remodeling at cellular and subcellular levels characteristic of HF can lead to action potential (AP) prolongation and delayed repolarization, which could be important causes of EAD formation. In turn, these changes could be linked to the modification in ion channel behavior, especially the L-type calcium channels (LTCC) whose activity is highly regulated via cAMP by various molecules co-localized in the T-tubular region, and the disruption of intracellular calcium (Ca^+2^) handling also observed in unhealthy myocytes (Tomaselli and Marbán, 1999; Lyon et al., 2009; Zima et al., 2014). These subcellular changes are often exacerbated by sympathetic stimulation which increases the levels of cAMP and can cause an increase in inward current, particularly through LTCCs. This, in turn, can destabilize the labile plateau phase of APs (Tomaselli and Zipes, 2004). However, the exact mechanism linking changes in T-tubule organization and in cAMP compartmentation to abnormal LTCC function and the development of arrhythmogenic triggers remains unknown.

L-type calcium channels play an important role in excitation–contraction coupling and influence the electrical and mechanical functioning of cardiac muscle (Bryant et al., 2015). In ventricular myocytes, under normal conditions, LTCCs are clustered in the transverse tubules (T-tubules). Gradual T-tubule loss, a known effect of HF, forces LTCCs to be redistributed to the surface membrane where they maintain their function but experience a different environment than that of the dyadic space (Sanchez-Alonso et al., 2016).

Within the T-tubules, LTCCs are usually associated with a number of specific macromolecular signaling complexes and scaffolding proteins which allow for precise control of Ca^+2^ signaling (Bers, 2008; Timofeyev et al., 2013). One such signaling complex is the β_2_AR, responsible for mediating the functional effects of cathecolamines in the heart, and more specifically, inside T-tubules, where they are confined. Selective sympathetic stimulation of the two receptor subtypes, β_1_AR and β_2_AR, lead to distinct physiological responses, based on their different localization and kinetics (Nikolaev et al., 2010). β_1_ARs stimulate the cAMP-dependent PKA-mediated phosphorylation of phospholamban and cardiac contractile proteins. Sympathetic stimulation of the β_2_AR, on the other hand, activates the G-protein/adenylyl cyclase (AC)/cAMP/PKA pathway which leads to the phosphorylation of several substrates, including LTCCs. In failing cardiomyocytes β_2_ARs are redistributed from the T-tubular membrane to the non-T-tubular membrane areas where stimulation of the receptor induces far reaching cAMP signals, similar to those elicited by β_1_AR stimulation (Nikolaev et al., 2010; Wright et al., 2014).

Apart from the molecules mentioned above, PDEs, which cleave cAMP into adenosine monophosphate (AMP) and protein phosphatases that dephosphorylate LTCCs also have an effect on the phosphorylation state of LTCCs. Furthermore, PDE molecules also show a high localization to the T-tubule membrane in healthy cells and along with AC and PKA play a key role in controlling the compartmentation of cAMP (Heijman et al., 2011). Activation of PKA by cAMP leads to PDE phosphorylation, increasing its activity, which in turn removes phosphorylation from other molecules. Thus, PDEs provide a negative feedback-loop for cAMP level control. Disrupting the localized PDE expression could lead to increased levels of cellular cAMP and inhibition of the PDE hydrolyzing effect (Wright et al., 2014).

The goal of this study was to uncover the mechanism by which HF-induced changes in cAMP compartmentation combines with the LTCCs T-tubule-to-surface-membrane redistribution to cause an abnormal LTCC current which promotes the development of EADs in human ventricular myocytes. To this end, we developed a novel model, using the O’Hara-Rudy human cardiac ventricular AP formulation (O’Hara et al., 2011) combined with the Heijman and colleagues β-adrenergic signaling simulations (Heijman et al., 2011) as our cornerstone. As the specific aim of this study was to dissect the effects of β_2_AR signaling localization loss and PDE-dependent cAMP level control impairment on the electrophysiological function of the cell, our single-cell model brought together various aspects of microdomain remodeling characteristic to HF, such as LTCC redistribution presented by Sanchez-Alonso et al. (2016), and modified cAMP compartmentation observed experimentally by Nikolaev et al. (2010). Our results show valuable insight into the pathophysiological changes that favor the development of cellular arrhythmogenic triggers in HF patients.

## Materials and Methods

### Overview of the Modeling Approach to Simulate the Behavior of Human HF Ventricular Myocyte

As a baseline model representing the healthy human ventricular myocyte, we used the O’Hara-Rudy human ventricular AP model (O’Hara et al., 2011) combined with the Heijman and coleagues β-adrenergic signaling model (Heijman et al., 2011), as described previously (O’Hara and Rudy, 2012). Within this model, the behavior of the LTCCs is described using a Hodgkin–Huxley formulation, and LTCC calcium dependent inactivation (CDI) depends on the concentration of Ca^+2^ the channels sense. To simulate a human HF ventricular myocyte, we incorporated various changes into the baseline model, reproducing ionic and structural remodeling observed *in vitro*, and described in detail in the sections below.

**FIGURE 1:**
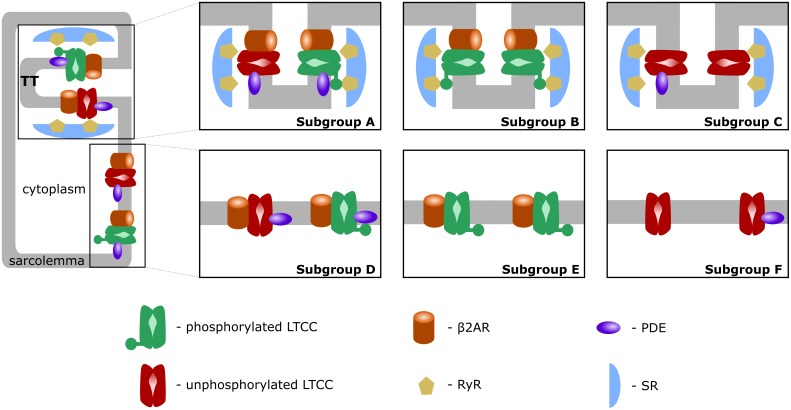
Schematic representation of LTCC subgroups A–F. LTCCs are located either in the T-tubular membrane (subgroups A–C) or in the bulk of the sarcolemma (subgroups D–F). LTCCs were either associated with both β_2_ARs and PDEs (subgroups A, D), solely with β_2_ARs (subgroups B, E), or not associated with β_2_ARs at all (subgroups C, F). The channels were either phosphorylated (red) or unphosphorylated (green).

First, similar to a previous study (Sanchez-Alonso et al., 2016), we introduced T-tubular microdomain loss, resulting in LTCCs and Na^+^/Ca^+2^ exchangers (NCXs) being redistributed from the T-tubular membrane to the bulk of the sarcolemma and in RyRs decoupling from LTCCs. However, instead of creating a single model of an HF ventricular myocyte as in the Sanchez-Alonso and colleagues study, we permitted the amount of microdomain loss to vary and thus created a family of HF myocyte models that allowed us to analyze different stages of the disease. The progression of the disease was also modeled by letting the phosphorylation level of the channels to vary based on the presence of β_2_ARs and PDEs in both the T-tubular space and in the bulk of the cytoplasm or sarcolemma. The more advanced the stage of the disease was, the more PDE molecules were redistributed from the dyadic space to the bulk of the cytoplasm, and the more β_2_ARs were shifted from the T-tubular to the surface membrane. Since LTCCs would display a different behavior based on their location and the signaling domain they experience, we divided LTCCs within a myocyte into six different subgroups (subgroups A–F) based on their position in the T-tubular or surface membrane and their signaling environment, where they may or may not experience the phosphorylating effect of PKA and the hydrolyzing effect of PDEs (Figure 1, subgroups A–F). The resulting models containing all LTCC populations were utilized to carry out computer simulations uncovering the mechanisms responsible for the generation of EADs in human HF myocytes under adrenergic stimulation.

### Introducing T-Tubular Mirodomain Degradation in HF

The combined O’Hara-Rudy and Heijman model contained LTCCs exclusively in the T-tubular membrane (O’Hara and Rudy, 2012). We used a similar approach to that in Sanchez-Alonso et al. (2016), and allowed for LTCCs to be redistributed to the surface membrane. Thus, in addition to the already defined dyadic volume, we included a sub-sarcolemmal volume which allowed for Ca^+2^ accumulation near the intracellular mouth of LTCCs in the surface membrane. We described fluxes from and into this volume in the O’Hara-Rudy myocyte model (O’Hara et al., 2011) based on the work of Shannon et al. (2004) and Grandi et al. (2010). Similar to Sanchez-Alonso and colleagues, we modeled the relocation of LTCCs to the surface membrane by allowing the redistributed channels to contribute to and sense the Ca^+2^ concentration from the sub-sarcolemmal volume as opposed to that from the dyadic volume for those remaining in the T-tubular membrane. However, to study the progression of the disease, we created multiple models in which T-tubule microdomains were gradually lost. In order to account for this HF-induced T-tubular degradation, we introduced the parameter f_TT in the model and used it to encode T-tubule integrity. The value of this parameter was allowed to range from fully intact (*f_TT* = 1.0, 0% T-tubule loss) to completely degraded (*f_TT* = 0.0, 100% T-tubule loss) in increments of 0.1. We then set the fraction of LTCCs that were redistributed from the T-tubule to the surface membrane to be directly proportional to the loss of T-tubules. Thus, when the T-tubular membrane was intact (f_TT = 1.0), all LTCCs were confined to it. In contrast, when the microdomain was completely disrupted (f_TT = 0.0), all LTCCs were redistributed to the surface membrane.

### Modeling the Effect of T-Tubular Loss on the Na^+^/Ca^+2^ Exchanger and RyRs

Two other molecules affected by the loss of T-tubular domains are the NCX molecules and RyRs. In the original O’Hara-Rudy model, RyRs are always associated with LTCCs from the T-tubular membrane and only a fifth of the NCX are located in the surface membrane, while the rest reside in the T-tubular subspace (O’Hara et al., 2011). We used the same approach as in Sanchez-Alonso and colleagues, and redistributed a fraction of NCXs proportional to the amount of de-tubulation from the T-tubular sites to the sarcolemma based on observations made by Gadeberg et al. (2016). The same fraction, but this time of RyRs, was also decoupled from LTCCs (Sanchez-Alonso et al., 2016).

### Representing the Effect of β_2_ARs and PDEs Presence on LTCCs

Activation of the β_2_AR leads to an increase in adenylyl cyclase (AC) activity which, in turn, increases the levels of cAMP. Next, cAMP binds to the regulatory subunits of PKA, enabling the catalytic subunits to phosphorylate their substrates at specific serine or threonine residues. This signaling cascade is counterbalanced by the hydrolyzing activity of PDEs that degrades cAMP (Heijman et al., 2011). Because there is only one detectable phosphorylation site that allows PKA to regulate LTCCs (Kamp and Hell, 2000), an LTCC can be characterized by one of two conditions: phosphorylated or not phosphorylated. In other words, an LTCC cannot be partially phosphorylated, and phosphorylation can be modeled as a binary process. Based on these observations, we divided all LTCCs within a myocyte into two different populations, phosphorylated and not phosphorylated.

We also introduced two new parameters, f_PDE and f_B2AR to quantify the fractions of LTCCs that experience phosphorylation due to the activation of the β_2_AR/G-protein/AC/cAMP/PKA signaling cascade and dephosphorylation due to PDE activity. In other words, localized upregulation of I_CaL_ due to β_2_AR stimulation was encoded to affect only a certain proportion of LTCCs, which varied from all LTCCs (f_B2AR = 1.0) to none (f_B2AR = 0.0) in increments of 0.1. Similarly, the hydrolyzing activity of PDEs was allowed to range from affecting no LTCC (f_PDE = 0.0), to affecting all LTCCs (f_PDE = 1.0) in steps of 0.1.

### Allowing LTCCs to Function in Different Locations and Signaling Domains

A comprehensive population-based approach in which LTCCs were distributed between the six different subgroups mentioned in the overview and described below was then used to integrate the effects of T-tubule loss and the disruption of β_2_AR and PDE signaling localization on the LTCC current in HF myocytes. The Hodgkin–Huxley formulation of LTCC current combines together the number of LTCCs and their conductance in order to obtain a current density for the whole cell. Empirically, no significant changes in LTCC current density have been observed in failing human cardiac myocytes (Piacentino et al., 2003; Chen et al., 2008), which may be due to HF myocytes showing a decrease in LTCC number, paired with an increase of the channel’s activity (Bryant et al., 2014). However, in our myocyte model the total number of LTCCs and their conductance were kept constant, each LTCC being assigned a current (I_CaL_) equal to unity. This assumption was made in order to limit the degrees of freedom of our model, to be able to draw mechanistic insights from the data obtained. If an LTCC was phosphorylated by PKA, the amplitude of the current through that channel was increased by a factor of 2.5 (Heijman et al., 2011). Subgroup A, depicted in Figure 1, subgroup A, contains channels identical to the LTCCs found in healthy myocytes: they are located in the T-tubular membrane and experience cAMP levels regulated by both PKA and PDE. In the second group – subgroup B (Figure 1, subgroup B), LTCCs located in the T-tubular membrane do not experience the hydrolyzing activity of PDE, so they are assumed to always be phosphorylated. In contrast, the subgroup C of LTCCs (Figure 1, subgroup C), which experience the activity of PDE, are assumed to never be phosphorylated as they lack association with β_2_ARs. Based on this logic, another subgroup could be defined as the channels lacking both PDE and β_2_AR in the T-tubular membrane, but physiologically, these would be indistinguishable from the channels in subgroups C as they would never be phosphorylated. Therefore, we assumed they are also part of subgroup C. The other three groups (subgroups D–F) are similar to the ones just mentioned but are located in the surface cellular membrane as opposed to the T-tubular membrane. The total LTCC current for a cell was calculated as the sum of the currents passing through each channel population.

### The Effects of Phosphorylation on Other Molecules

A similar population-based approach was used to represent the degree of PKA phosphorylation, in the presence or absence of β_2_AR stimulation, of seven different targets: RyR, Phospholamban (PLB), Slow Delayed Rectifier K^+^ Current (I_Ks_), Fast Na^+^ Current (I_Na_), Na^+^/K^+^ATPase Current (I_NaK_), rapidly activating K^+^ current (I_Kur_) and Troponin I (TnI).

Assuming that channel gating has no effect on phosphorylation, the molecules mentioned above can have only two distinct conformations (phosphorylated and non-phosphorylated). Therefore, each of the seven phosphorylation targets were divided into two subgroups: phosphorylated and not phosphorylated. The effect of phosphorylation on each target, as described by Heijman et al. (2011), was included in the model. Basal conditions were modeled to account for the phosphorylation of 25% of targets while β_2_AR stimulation led to the phosphorylation of 75% of each substrate population. These values were chosen in order to obtain LTCC current traces in the healthy myocytes similar to those experimentally recorded and presented by Bryant et al. (2014). In order to be as physiologically accurate as possible, the model also accounted for CaMKII-dependent phosphorylation of I_CaL_, I_Na_ and late I_Na_ (I_Na,L_) based on recently published data (Sanchez-Alonso et al., 2016). For each of these molecules it was assumed that PKA and CaMKII phosphorylation are independent processes. Therefore, the targets were divided into four populations. At each time step, the currents through the LTCCs and I_Na_ channels were computed for both (phosphorylated and non-phosphorylated) populations. The total current was obtained by adding together the currents through each population and thus integrating the β_2_AR and CaMKII cascades. While the level of PKA phosphorylation varied for the purpose of this study, the CaMKII phosphorylation level was kept constant.

### I_CaL_ and Action Potential Duration (APD) Analysis Protocol

**FIGURE 2:**
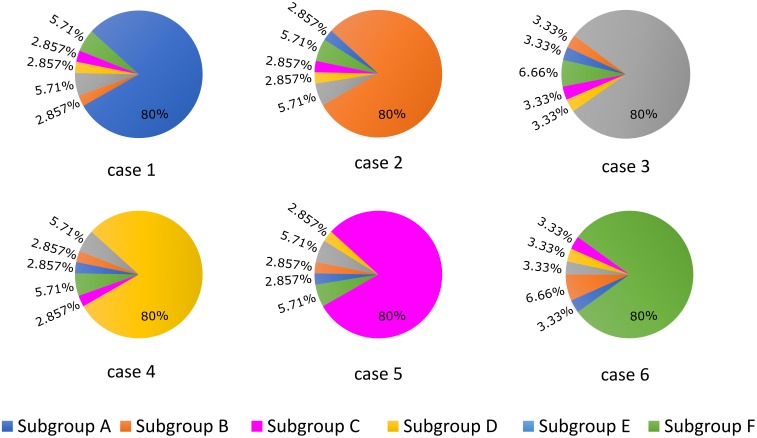
Schematic representation of the LTCC distribution between the six different subgroups (A–F) for each case analyzed (1–6). The total number of LTCCs in the cells from each case was kept constant, but the channels were divided into the six subgroups in the ratios displayed. In case 1, 80% of the channels are located inside the T-tubular membrane, while the remaining ones were distributed to the remaining subgroups based on their probability to be associated with PDEs and β_2_ARs. In case 2, the dominant channel population was represented by channels located in T-tubules, lacking PDE association, while in case 3, the majority of channels were also located in the T-tubular membrane, but were not associated with β_2_ARs. Cases 4, 5, and 6 are similar to cases 1, 2, and 3, but their dominant channel populations are located in the surface sarcolemma.

APD_90_ was computed as the time point of 90% repolarization of the membrane potential minus the time point of maximal upstroke velocity (dV/dt)_max_. For the first part of the study, we established six different cases (cases 1–6) in which 80% of LTCCs within a cell were assigned to one of each of the six subgroups described above and in Figure 1, and for each case the remaining channels were distributed between the other five subgroups (Figure 2). This allowed us to observe and analyze separately the effects of LTCC redistribution, loss of β_2_AR signaling localization, and PDE’s impaired control of cAMP on the LTCC current and APD_90_. The model myocyte was first allowed to reach steady state under a voltage clamp of -96.7 mV. To determine the effect of each parameter (f_TT, f_B2AR, and f_PDE) on the LTCC current, the membrane voltage was then stepped up from -96.7 to -6.7 mV, clamped again, and the LTCC current was recorded. In order to analyze the effect of the same parameters on APD_90_, the myocyte was paced at near resting pacing rates (1000 ms, 60 bpm) until steady state was reached; the following three beats were recorded.

### Protocol for Evaluating EADs in the Human Ventricular AP Model

The same protocol used to analyze APD_90_, was also used to investigate how the model variables drive the emergence of EADs. The myocyte was again paced at near resting pacing rates (1000 ms, 60 bpm) until steady state was reached and the following 15 beats were recorded. For this part of the study, LTCCs were distributed into the six different subgroups represented in Figure 1 by varying the model variables f_TT, f_B2AR, and f_PDE. The probability that an LTCC was corresponding to one of the subgroups was computed by multiplying these state variables. The procedure was repeated for values of f_TT and f_PDE uniformly covering the range of possible values from 0.0 to 1.0 in increments of 0.1, and for values of the f_B2AR ranging from 0.0 to 1.0 in increments of 0.25. The APs generated both in the presence and absence of adrenergic stimulation were analyzed.

## Results

### HF Remodeling Leads to a Higher Amplitude, Longer Lasting LTCC Current

**FIGURE 3:**
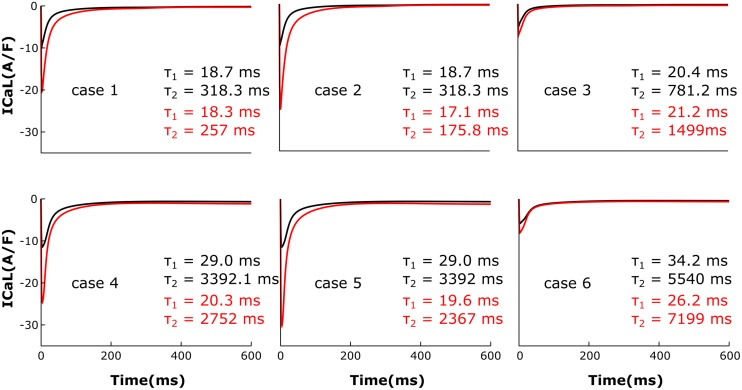
The effect of microstructure remodeling on LTCC current. Total LTCC current was plotted over time from cells corresponding to cases 1–6 described in methods, under both basal (black) and sympathetic stimulation (red) conditions. The decay time constants for the current through the channels in the T-tubules and the ones in the bulk of the sarcolemma were displayed as τ_1_ and τ_2_.

The LTCC current traces from the cases outlined in Figure 2 are plotted in Figure 3 under both basal and adrenergic stimulation conditions. The control case (Figure 3, case 1 – black) was represented by the LTCC current in the healthy myocyte with intact tubulation as well as localization of β_2_AR and PDE activity. LTCC current decay was rapid, especially in the T-tubules (τ_1_ = 18.7 ms, τ_2_ = 318.3 ms), due to the strong CDI caused by the accumulation of Ca^+2^ in the dyadic volume of the model. β_2_AR stimulation in the control case led to an increase in the magnitude of LTCC current and a faster decay rate (τ_1_ = 18.3 ms, τ_2_ = 257 ms), as compared to the basal conditions (Figure 3, case 1 – red). The effect of PDE on controlling cAMP levels and thus LTCCs level of phosphorylation was evaluated by removing PDE from the model (Figure 3, case 2). Although this change had little to no effect for the case with basal PKA phosphorylation (black), it considerably increased LTCC current peak when adrenergic stimulation was applied (red). This showed that PDE has a protective role that helps lower inward LTCC current, as PKA activity was not inhibited at all by the hydrolyzing activity of PDE on cAMP. Removing all β_2_ARs led to smaller currents as PKA did not phosphorylate any channel (Figure 3, case 3). In the control case, when tubulation was removed and LTCCs shifted to the surface membrane (Figure 3, case 4), Ca^+2^ was not able to accumulate in significant amounts in the proximity of the LTCCs. Thus CDI was delayed, peak current was elevated, and I_CaL_ inactivation rate was lower (τ_1_ = 29.0 ms, τ_2_ = 3392 ms). Applying β_2_AR stimulation in this case led to an even larger in magnitude and slower current (Figure 3, case 4 – red), and removing PDE further exacerbated this effect (Figure 3, case 5). Removing T-tubules when β_2_ARs were absent (Figure 3, case 6) led to a slower decaying current, of similar magnitude to that in fully tubulated cells. Overall, subcellular changes associated with the HF phenotype, such as de-tubulation, LTCC redistribution to the surface membrane, and loss of β_2_AR and PDE signaling localization, led to an increase of the LTCC peak current and a delay in the channels’ inactivation.

### HF Remodeling Leads to Altered SR Ca Release and Ca Transient

**FIGURE 4:**
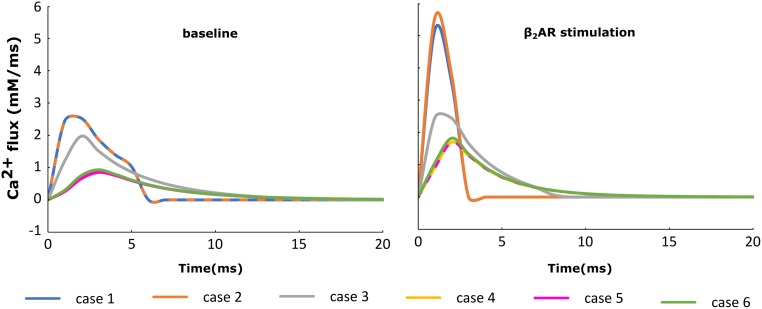
The effect of microstructure remodeling on SR Ca^+2^ release. The Ca^+2^ flux from the SR into the cytoplasm via RyRs was plotted over time from cells corresponding to cases 1–6, under both basal **(left)** and sympathetic stimulation **(right)** conditions.

**FIGURE 5:**
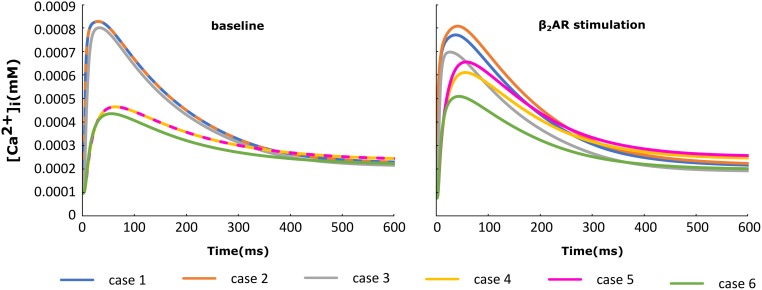
The effect of microstructure remodeling on Ca^+2^ transient. The Ca^+2^ concentration inside the cytoplasm was plotted over time from cells corresponding to cases 1–6 described in methods, under both basal **(left)** and sympathetic stimulation **(right)** conditions.

For each of the cases described above, the Ca^+2^ flux from the SR into the cytoplasm through RyRs is plotted in Figure 4 and the Ca^+2^ concentration inside the cytoplasm in Figure 5. When adrenergic stimulation is applied, the β_2_AR/G-protein/AC/cAMP/PKA signaling cascade is triggered, which leads to the phosphorylation of RyRs along with LTCCs. Thus, not only do RyRs experience a higher Ca^+2^ concentration in the dyadic space due to the phosphorylation of LTCCs, but their activity is also enhanced by direct PKA phosphorylation, leading to a higher Ca^+2^ flux from the SR into the cytoplasm (Figure 4). However, due to the higher Ca^+2^ concentration inside the dyadic space, RyRs deactivate faster under adrenergic conditions, and the Ca^+2^ concentration inside the cytoplasm does not differ greatly between baseline and adrenergic stimulation conditions in intact myocytes (Figure 5). The β_2_AR enhancing effect on Ca^+2^ release appears stronger when the microdomain is degraded. By removing T-tubules, we disrupt the association between LTCCs and RyRs within the dyadic space. Removing T-tubules and relocating LTCCs from the dyadic space to the surface of the membrane leads to a lower Ca^+2^ concentration within the dyadic volume, and a weaker release of Ca^+2^ from the SR into the cytoplasm. While completely removing β_2_AR molecules in the intact HF myocyte model leads to a lower amount of Ca^+2^ being released via RyRs in, it has the opposite effect in de-tubulated cells (Figure 4). This is explained by the fact that LTCCs in the surface membrane experience CaMKII phosphorylation, while those in the T-tubular membrane do not (Sanchez-Alonso et al., 2016). Overall, the Ca^+2^ concentration inside intact myocytes is higher than that in de-tubulated cells due to higher LTCC current through T-tubular channels, and an increase in the flux of Ca from the SR into the cytoplasm.

### Changes in I_CaL_ and RyR Function Impact the NCX Current

**FIGURE 6:**
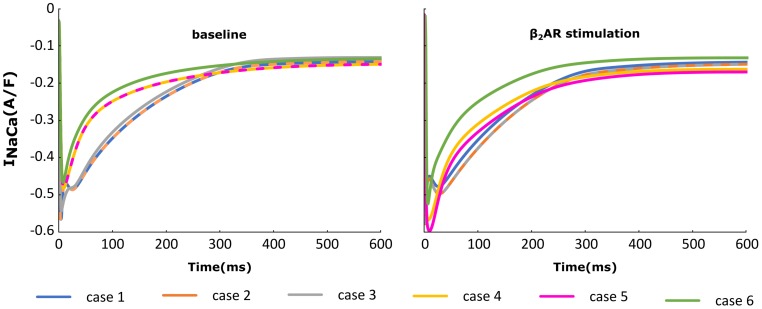
The effect of microstructure remodeling on I_NaCa_ current. The I_NaCa_ current was plotted over time from cells corresponding to cases 1–6, under both basal **(left)** and sympathetic stimulation **(right)** conditions.

The NCX (INaCa) current traces from the cases outlined in Figure 2 are plotted in Figure 6 under both basal and adrenergic stimulation conditions. Similar to the re-distributed LTCCs, NCX molecules which shifted to the surface membrane experience the Ca^+2^ concentration from the sub-sarcolemmal volume. Under baseline conditions, microdomain loss lead to a small decrease in I_NaCa_ corresponding to a lower SR Ca release and an overall lower Ca^+2^ transient. However, similar to I_CaL_, under adrenergic stimulation I_NaCa_ displayed an increase in amplitude when the myocyte underwent de-tubulation, but not a slowdown in decay rate. These results can be explained based on the observations we made before on RyR and LTCC function. NCX is an antiport driven by the concentration gradients of Ca^+2^ and Sodium. A high amplitude I_CaL_ within a de-tubulated cell leads to a high concentration of Ca inside the sub-sarcolemmal volume which in turn causes a steep gradient between the inside and outside of the cell. Thus, we observe a high amplitude I_NaCa_ under adrenergic stimulation in the HF myocyte, which in theory should lead to APD shortening.

### Abnormal LTCC Current Affects APD_90_

**FIGURE 7:**
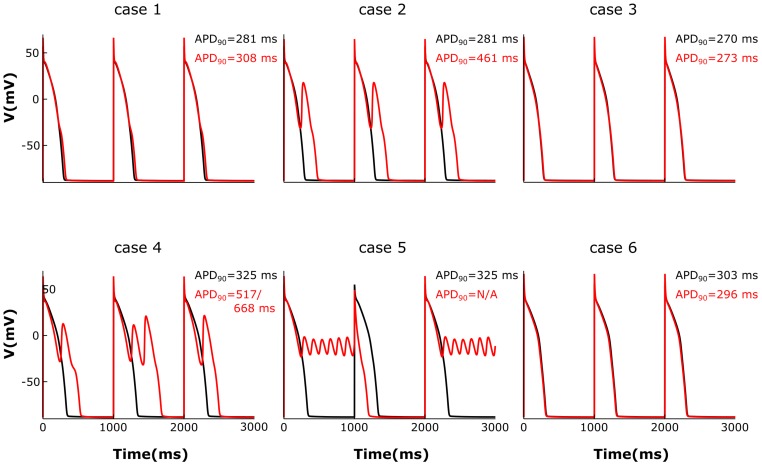
The effect of microstructure remodeling on membrane voltage and APs. The membrane potential of cells from cases 1–6 is plotted over time under both basal (black) and sympathetic stimulation (red) conditions. EADs emerged only under sympathetic stimulation in the cells where most LTCCs were either located in the T-tubules and lacking PDEs **(case 2)**, or they were in the surface membrane and experienced the effect of both β_2_ARs and PDEs **(case 4)**. When most LTCCs were located in the bulk of the sarcolemma and were not associated with PDEs **(case 5)**, the cells were not able to repolarize and remained in an oscillatory state until the next stimulus was applied.

We then analyzed the effect of varying the parameters encoding for T-tubule integrity, β_2_AR and PDE on AP shape and duration. The membrane potential corresponding to the six cases described above was plotted in Figure 7. For the control case under no adrenergic stimulation, an APD_90_ of 281 ms was recorded. Adding β_2_AR stimulation lead to an increase and slower decay rate in the LTCC current, which caused a slight prolongation of APD_90_ to 308 ms (Figure 7, case 1). Removing PDEs from the model and applying β_2_AR stimulation caused the emergence of single EADs in intact myocytes (Figure 7, case 2). Removing all β_2_ARs from the model, on the other hand, had the opposite effect, preventing EADs and causing APD_90_ to shorten (Figure 7, case 3). When tubulation was removed from the control case, and no adrenergic stimulation applied, the observed increase in LTCC current led to a corresponding 44 ms APD_90_ prolongation.

The effect of β_2_-stimulaton was significantly stronger in the absence of T-tubules resulting in an APD_90_ of 517/668 ms and the development of multiple EADs (Figure 7, case 4). However, in de-tubulated myocytes, applying adrenergic stimulation while lacking PDE led to an oscillatory state in which the cells did not repolarize before the next stimulus was applied (Figure 7, case 5). Removing all β_2_ARs from the de-tubulated model prevented EAD formation (Figure 7, case 6).

### Effects of De-tubulation and β_2_AR Loss on APD_90_

**FIGURE 8:**
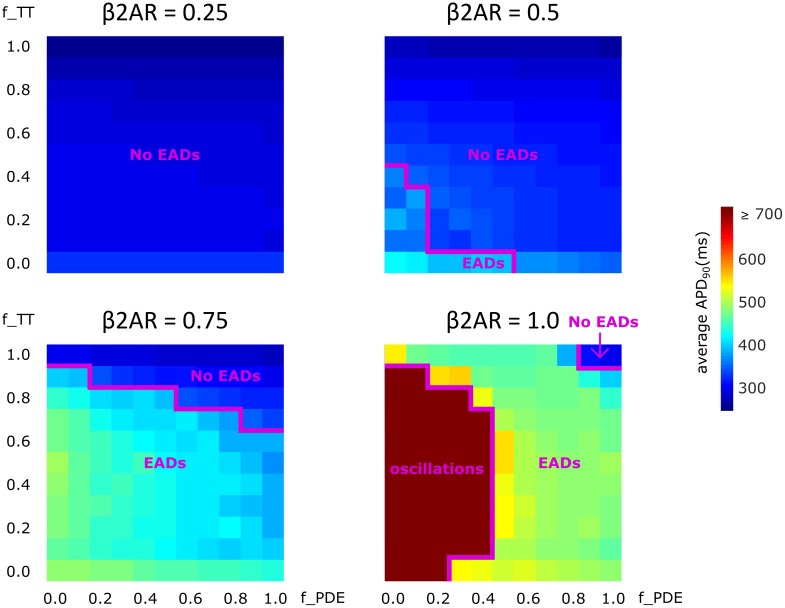
The effect of microstructure remodeling on APD_90_ and EAD emergence. Heatmaps of the average APD_90_ under sympathetic stimulation for four fixed values of the f_B2AR parameter. The cells range from intact (f_TT = 1.0) to fully de-tubulated (f_TT = 0.0), and PDEs range from present and fully functional (f_PDE = 1.0) to completely absent (f_PDE = 0.0). EAD occurrence and oscillation behavior were also represented.

Lastly, we analyzed how gradual changes in the amount of de-tubulation as well as β_2_AR and PDE signaling localization affect APD_90_ and EAD development. The heatmaps in Figure 8 were plotted in order to quantify how each of these variables impacts APD_90_ and EAD formation. The results demonstrate that EADs occurred only under adrenergic stimulation. Moreover, the loss of PDE control of cAMP in the dyadic space also contributed to the emergence of EADs.

No EADs formed when only 25% of the β_2_ARs were present. When 50% of the β_2_ARs were present EADs emerged either in fully de-tubulated cells, or in cells that lacked more than 20% of their PDE. In all the other cells, APDs appeared to increase proportionally to PDE and tubulation loss but no arrhythmogenic triggers were observed. The same trend was detected when the amount of functional β_2_AR molecules was increased to 75%. In this case, we observed more frequent EAD emergence, while the presence of intact tubulation and PDE had a protective effect. When all β_2_ARs were present and functional, the model generated membrane potential oscillations for most cells that were lacking more than 60% of their initial T-tubules. EADs were obtained in almost all other cases, except the one in which tubulation was intact and at least 90% of PDEs were present. Thus, the presence of PDE molecules had a protecting effect, opposing APD_90_ prolongation and in some cases inhibiting EAD development.

## Discussion

### Findings and Significance

Because HF electrophysiological remodeling is such a complex phenomenon (Tomaselli and Marbán, 1999), for the purpose of this computational study we used simulations to model and integrate only the structural changes described above. There are multiple other aspects described in Limitations that could be introduced in subsequent studies. Thus, the goal of this project was to elucidate the mechanisms by which subcellular changes in cardiac myocytes lead, via altered LTCC function, and under adrenergic stimulation, to the generation of cellular-level triggers of arrhythmia in human HF. To accomplish this goal, we developed a new ventricular myocyte computational approach that integrated various known characteristics of HF and allowed us to vary their severity. The model included the effects of microdomain remodeling on LTCC as in Sanchez-Alonso et al. (2016), as well as novel developments incorporating β_2_AR stimulation, and arrhythmogenic remodeling of β_2_ adrenergic signaling, as well as impaired control of cAMP levels due to altered PDE localization. We found that redistribution of the LTCCs combined with an enhanced phosphorylation of the channel due to abnormally high cAMP levels caused increases in LTCC current magnitude and duration. This resulted in AP prolongation and the development of single EADs, multiple EADs, or oscillations, depending on the amount of remodeling present.

### Transverse Tubules Loss in HF

The T-tubule system plays a vital role in normal ventricular cell function. Its structure and distribution are responsible for the rapid electric excitation, and the initiation and synchronous triggering of Ca^+2^ release from the sarcoplasmic reticulum (SR). The increase in Ca^+2^ levels throughout the cytoplasm leads to the coordinated contraction of all contractile units inside a cell (Wei et al., 2010). Scanning ion conductance microscopy (SICM) and fluorescence staining experiments have shown that ventricular myocytes obtained from HF patients are characterized by a decrease in T-tubule density (Lyon et al., 2009; Wei et al., 2010). This change in surface topography has been shown to take place gradually as the cardiac muscle transitions from hypertrophy to HF and to have a negative impact of excitation–contraction coupling (Wei et al., 2010). Song and colleagues proposed that this deleterious effect is due to the reorganization of subcellular microdomains, which force RyRs to decouple from their partner LTCC and become orphaned. Thus Ca^+2^ ions flowing through the LTCCs fail to bind to RyRs and do not cause further Ca^+2^ release from the SR (Song et al., 2006). Other studies have shown that in HF, the loss of T-tubule microdomains is usually complemented by a spatial redistribution of LTCCs from the T-tubule membrane to the bulk of the sarcolemma, where they experience a different signaling environment (Sanchez-Alonso et al., 2016). Finally, work by Kawai et al. (1999) and Bryant et al. (2014) showed that complete myocyte de-tubulation is associated with reduction in I_CaL_ and shortening of APD. Taken together, the findings of these previous experimental studies suggest that altered LTCC function is a likely candidate for the mechanism linking T-tubule microdomain loss to the emergence of arrhythmogenic triggers in ventricular HF myocytes.

A novel aspect of our simulation approach lies in the fact that instead of creating a single model, by varying the amount of remodeling we obtained a family of models which allowed us to get an accurate depiction of the implications of T-tubule and cAMP compartmentalization loss in HF. By using this approach, we were able to analyze the effects of T-tubular loss alone, as well as in conjunction with a few other pathophysiological changes previously observed in HF in a setting that resembled the progression of the disease. Because our study showed an increase in I_CaL_ and APD due to de-tubulation, it appears that our results are at odds with the results by Kawai et al. (1999) and Bryant et al. (2015). However, that is not the case. The difference in our results comes mainly from the way we define de-tubulation and the exact distribution of channels between the T-tubular and the bulk of the sarcolemma. In our study, we view T-tubular microdomain disruption as a gradual process which slowly advances as HF progresses, similar to the changes that the myocytes from the above-mentioned studies undergo after coronary artery ligation (CAL). We are not trying to reproduce the data from a specific phase of the disease, as that would be impossible without introducing all the aspects of HF electrophysiological remodeling, but rather analyze the specific changes caused by gradual de-tubulation and cAMP re-distribution. If we compare one of our myocyte models constructed using the same ratio of channels in the T-tubular membrane vs. the bulk of the sarcolemma as in the CAL myocytes from the Bryant and colleagues, with what would be the equivalent of a healthy human myocyte in the Sanchez-Alonso and colleagues study (Sanchez-Alonso et al., 2016), we get similar whole cell I_CaL_ current density (data not shown). However, because in their study de-tubulation is defined as a fast, chemical process which completely removes all T-tubular microdomain, and in our model de-tubulation is a slow process which allows the channels to relocate to the surface membrane, our results are different when we analyze de-tubulation. We could partially replicate their results if we completely eliminated the T-tubular component of I_CaL_.

Furthermore, even though our results show an increase in I_CaL_ density due to HF when there are no experimental studies in humans showing that, we have to keep in mind that I_CaL_ peak and density seem to vary in HF from study to study (Tomaselli and Marbán, 1999), and *in-silico* studies don’t always match the experimental ones (Elshrif et al., 2015) when it comes to this issue. However, our results do reflect the most common change in I_CaL_ morphology caused by HF which is a slowing of the decay of the whole-cell current. Thus, our findings are consistent with the findings described above. They reproduce the change in I_CaL_ shape, add new insight about different stages of the disease, and highlight the existence of a threshold of T-tubule integrity that can predict the development of cellular level triggers of arrhythmia, as seen in Figure 8.

### The Role of β_2_AR and PDE Signaling Localization Loss in EAD Development

Another hallmark of HF is βAR desensitization. Wright and colleagues showed, by combining a nanoscale SICM with a fluorescence resonance energy transfer (FRET) approach, that in healthy ventricular myocytes β_2_AR-induced cAMP signals were highly localized within the T-tubular domain (Wright et al., 2014). Heijman and colleagues used a computational model to describe in great detail the quantitative contribution of various molecules, including LTCCs, and the effects of the above mentioned signaling localization in adrenergic stimulation (Heijman et al., 2011). In HF myocytes, sympathetic stimulation of β_2_AR appears to result in diffused, far reaching cAMP signals, which resembled those obtained via β_1_AR stimulation. This suggests that similar to LTCCs, β_2_ARs also change location from the T-tubular membrane to the surface membrane when tubulation is lost (Nikolaev et al., 2010). In addition, Lang and colleagues demonstrated that during end-stage HF, β_2_AR stimulation leads to heterogeneities in APD and arrhythmogenic activity (Lang et al., 2015). In our study, we were able to control the degree of both PDE and β_2_AR loss of function and redistribution and to analyze how it influences the electrophysiological functioning of the myocytes in HF, and specifically, LTCC function. Completely blocking β_2_AR function in the model did not lead to abnormal LTCC current, nor to EAD occurrence. This is consistent with the fact that ligand binding failed to trigger a signaling cascade, and LTCCs were never phosphorylated (βAR desensitization). It also explains why adrenergic blockers work well as antiarrhythmic agents. When the β_2_AR molecules were allowed to maintain partial functionality, but lost their localization, significant increases in LTCC current magnitude and APD_90_ as well as EAD development were observed. Moreover, when all β_2_AR molecules were present, instead of EADs, the cells displayed an oscillatory behavior in which the membrane potential did not return to its baseline value. This is a biological phenomenon also recorded in isoprenaline perfused rabbit ventricular cardiomyocytes affected by long QT syndrome and thought to lead to sudden cardiac death (Liu et al., 2012). These changes were opposed by the presence of PDE, which appears to have a protective, anti-arrhythmogenic effect. The new mechanistic insights presented here show that the binding of the ligand to functional, redistributed β_2_ARs causes a cAMP signal propagation and amplification throughout the cell, which leads to the phosphorylation of multiple LTCCs across the cell membrane. Phosphorylated LTCCs have a higher open probability, which leads to a higher magnitude LTCC current, causing APD_90_ prolongation and EAD development.

## Conclusion

Altered LTCC current is a major factor in the process linking microstructural remodeling to the increased risk of cellular arrhythmia triggers development in HF patients. Our results show that the disruption of subcellular microdomains and the redistribution of LTCCs, PDEs, and β_2_ARs cause a higher-magnitude and longer-lasting LTCC current, which delays cellular repolarization. APs are thus prolonged and upon sympathetic stimulation, EADs may arise. These findings provide important insight into the mechanism underlying the development of cellular level triggers of arrhythmia in human HF and suggest β_2_ARs and PDEs as possible targets for future therapies and preventive treatments of ventricular arrhythmia.

### Limitations

Our study has several limitations. HF remodeling is a very complex process with many unknowns. Our model thus had various assumptions. First, in the lack of experimental evidence otherwise, we assumed that the LTCCs remaining in the T-tubular membrane in HF are fully functional; even super-resolution scanning patch clamp studies such as the one presented by Sanchez-Alonso et al. (2016) cannot measure LTCC activity deep inside the T-tubule system. Secondly, in order to be able to draw mechanistic insight, we limited the degrees of freedom of our model by keeping both the number of LTCCs as well as their conductance constant. This may be at odds with observations by Bryant and colleagues which suggest that in HF, myocytes show a decrease in the number of LTCCs and an increase in their activity (Bryant et al., 2014). Also, our model focused exclusively on the activity of LTCC channels in HF, and did not include changes in other currents such as I_Na,L_, I_Ks_, and I_Kr_ which are also affected by HF remodeling. Due to limited data on PKA-mediated phosphorylation of LTCCs in HF, we had to make approximations regarding the fraction of channels phosphorylated under baseline conditions and under adrenergic stimulation. Moreover, our study focuses exclusively on the effect of microdomain loss on β_2_AR, based on previous studies showing that HF leads to the re-distribution of these receptors which in turn affects the activity of LTCCs (Nikolaev et al., 2010), although β_1_AR stimulation is the major receptor mediating the effects of sympathetic stimulation of the heart. This could be explored in greater depth in future work. CaMKII-dependent phosphorylation of different channels was also included in the model, but as our study focused solely on the effect of sympathetic stimulation and PKA-dependent phosphorylation of LTCCs, CaMKII remodeling was not implemented. Since PDE acts as a negative feedback-loop for cAMP level control, the model assumed that PDE-mediated cAMP degradation is a process activated by PKA and thus PDE should only be active under sympathetic stimulation when PKA levels are increased. Lastly, the present study focused on single cell behavior; thus, it is possible that the results can be modulated by the effect of cell coupling on the susceptibility of cells to form EADs, as described by Xie et al. (2010).

## Author Contributions

AL helped design the experiments, performed the simulations, and wrote the manuscript. TO guided the design of the experiments. NT supervised the project and edited the manuscript.

## Conflict of Interest Statement

The authors declare that the research was conducted in the absence of any commercial or financial relationships that could be construed as a potential conflict of interest. The handling Editor and reviewer AG declared their involvement as co-editors in the Research Topic, and confirm the absence of any other collaboration.
